# Exploratory radiomic features from integrated ^18^F-fluorodeoxyglucose positron emission tomography/magnetic resonance imaging are associated with contemporaneous metastases in oesophageal/gastroesophageal cancer

**DOI:** 10.1007/s00259-019-04306-7

**Published:** 2019-03-27

**Authors:** Serena Baiocco, Bert-Ram Sah, Andrew Mallia, Christian Kelly-Morland, Radhouene Neji, J. James Stirling, Sami Jeljeli, Alessandro Bevilacqua, Gary J. R. Cook, Vicky Goh

**Affiliations:** 1 Department of Cancer Imaging, School of Biomedical Engineering and Imaging Sciences, King’s College London, London, UK; 2 Advanced Research Center for Electronic Systems (ARCES), University of Bologna, Bologna, Italy; 3 Department of Electrical, Electronic and Information Engineering “Guglielmo Marconi” (DEI), University of Bologna, Bologna, Italy; 4 King’s College London & Guy’s and St Thomas’ PET Centre, St Thomas’ Hospital, London, UK; 5 MR Research Collaborations, Siemens Healthcare, Frimley, UK; 6 Department of Computer Science and Engineering (DISI), University of Bologna, Bologna, Italy; 7 Cancer Imaging, School of Biomedical Engineering and Imaging Sciences, Lambeth Wing, St Thomas Hospital, Westminster Bridge Road, London, SE1 7EH UK

**Keywords:** ^18^F-fluorodeoxyglucose positron emission tomography/magnetic resonance imaging, Oesophageal cancer, Radiomic analysis

## Abstract

**Purpose:**

The purpose of this study was to determine if ^18^F-fluorodeoxyglucose positron emission tomography/magnetic resonance imaging (^18^F-FDG PET/MRI) features are associated with contemporaneous metastases in patients with oesophageal/gastroesophageal cancer.

**Methods:**

Following IRB approval and informed consent, patients underwent a staging PET/MRI following ^18^F-FDG injection (326 ± 28 MBq) and 156 ± 23 min uptake time. First-order histogram and second-order grey level co-occurrence matrix features were computed for PET standardized uptake value (SUV) and MRI T1-W, T2-W, diffusion weighted (DWI) and apparent diffusion coefficient (ADC) images for the whole tumour volume. K-means clustering assessed the correlation of feature-pairs with metastases. Multivariate analysis of variance (MANOVA) was performed to assess the statistical separability of the groups identified by feature-pairs. Sensitivity (SN), specificity (SP), positive predictive value (PPV), negative predictive value (NPV), and accuracy (ACC) were calculated for these features and compared with SUV_max_, ADC_mean_ and maximum diameter alone for predicting contemporaneous metastases.

**Results:**

Twenty patients (18 males, 2 female; median 67 years, range 52–86) comprised the final study cohort; ten patients had metastases. Lower second-order SUV entropy combined with higher second-order ADC entropy were the best feature-pair for discriminating metastatic patients, MANOVA *p* value <0.001 (SN = 80%, SP = 80%, PPV = 80%, NPV = 80%, ACC = 80%). SUV_max_ (SN = 30%, SP = 80%, PPV = 60%, NPV = 53%, ACC = 55%), ADC_mean_ (SN = 20%, SP = 70%, PPV = 40%, NPV = 47%, ACC = 45%) and tumour maximum diameter (SN = 10%, SP = 90%, PPV = 50%, NPV = 50%, ACC = 50%) had poorer sensitivity and accuracy.

**Conclusion:**

High ADC entropy combined with low SUV entropy is associated with a higher prevalence of metastases and a promising initial signature for future study.

**Electronic supplementary material:**

The online version of this article (10.1007/s00259-019-04306-7) contains supplementary material, which is available to authorized users.

## Introduction

Oesophageal/gastroesophageal (GOJ) cancer is a leading cause of cancer deaths worldwide with 572,034 new cases annually [1]. Surgery combined with neoadjuvant chemotherapy or chemoradiotherapy offers the best chance of cure. Data from the OEO2 [2] and MAGIC [3] trials for GOJ cancer have shown a 6 and 13% improvement in 5-year overall survival for neoadjuvant chemotherapy, respectively; while the CROSS trial [4] found a superior overall survival of 49 versus 24 months for neoadjuvant chemoradiotherapy plus surgery versus surgery alone. Despite this, overall survival remains poor, namely, the 5-year relative survival rate drops from 43 to 5% for localized and metastatic disease, respectively (https://www.cancer.net/cancer-types/esophageal-cancer/statistics).

Better patient stratification for treatment beyond our current staging practice remains a key challenge for GOJ patients given that quality of life remains poor for many patients post-surgery, taking up to 3 years to return to pre-therapy levels [5]. ^18^F-fluorodeoxyglucose positron emission tomography/magnetic resonance imaging (^18^F-FDG-PET/MRI) has shown promise as a one-stop imaging modality for oesophageal cancer [6]. A retrospective study of sequential ^18^F-FDG-PET/MRI of 19 patients with non-metastatic oesophageal cancer, comparing the diagnostic efficacy of endoscopic ultrasonography (EUS), computed tomography (CT) and ^18^F-FDG-PET/MRI for locoregional staging with a pathological reference standard in 15 patients found similar T-staging accuracy and slightly superior N-staging compared to EUS [6].

^18^F-FDG-PET/MRI also provides an opportunity to improve imaging phenotyping by combining molecular, functional and anatomical characteristics, lending itself to radiomic approaches to improve PET/MRI data integration [7]. Recent genomic analyses have highlighted genetic heterogeneity as an underlying cause for the differences in therapy outcomes [8]. We hypothesised that tumours with metastatic potential would demonstrate greater phenotypic heterogeneity. Thus, we aimed in the first instance to explore if any radiomic imaging features derived from ^18^F-FDG-PET/MRI are associated with metastases at staging in patients with GOJ cancer.

## Materials and methods

### Patient enrolment

Following institutional review board approval and informed consent, 24 prospective patients with newly diagnosed, histologically proven GOJ cancer were recruited from 2015 to 2018. None of the patients had a history of previous malignancy. All patients had undergone standard staging investigations that included endoscopic ultrasound (EUS), contrast-enhanced computed tomography (CT) of the thorax and abdomen and ^18^F-FDG PET/CT, and their final staging was documented in a multidisciplinary team (tumour review board) meeting.

### Image acquisition

Patients were injected with 326 ± 28 MBq of ^18^F-FDG following a 4–6 h fast, and blood glucose levels were verified as ≤10.0 mmol/L. PET/MRI examinations were performed on an integrated PET/MRI system (Biograph mMR, Siemens Healthcare, Erlangen, Germany) immediately following a clinical PET/CT acquisition. The mean ± SD time between injection of ^18^F-FDG and integrated PET/MRI examination was 156 ± 23 min. The PET/MRI was acquired from the skull base to mid-thigh (comprising 4–5 bed positions depending on patient height; 6 min per bed position). For each bed position a 2-point Dixon Volumetric Interpolated Breath-holdExamination (VIBE) sequence was applied to derive an attenuation map (μ-map) based on four tissue types: air, lung, soft-tissue and fat [9]. Other sequences per bed position included: T1-weighted Dixon VIBE (in-phase, out-of-phase, fat, water images), T2-weighted Half-Fourier-Acquired Single-shot Turbo spin Echo (HASTE) and free breathing diffusion-weighted sequences (DWI, *b* values of 50 and 900 s/mm^2^). No oral contrast was administered.

### Tumour node metastasis (TNM) staging

TNM staging was assigned as per the American Joint Committee on Cancer (AJCC) staging system (7th edition). Final TNM stage was defined by all standard staging investigations (not including ^18^F-FDG PET/MRI), documented in a multidisciplinary tumour board meeting, and used to categorize patients with metastatic versus non-metastatic disease.

### Image analysis

**Fig. 1:**
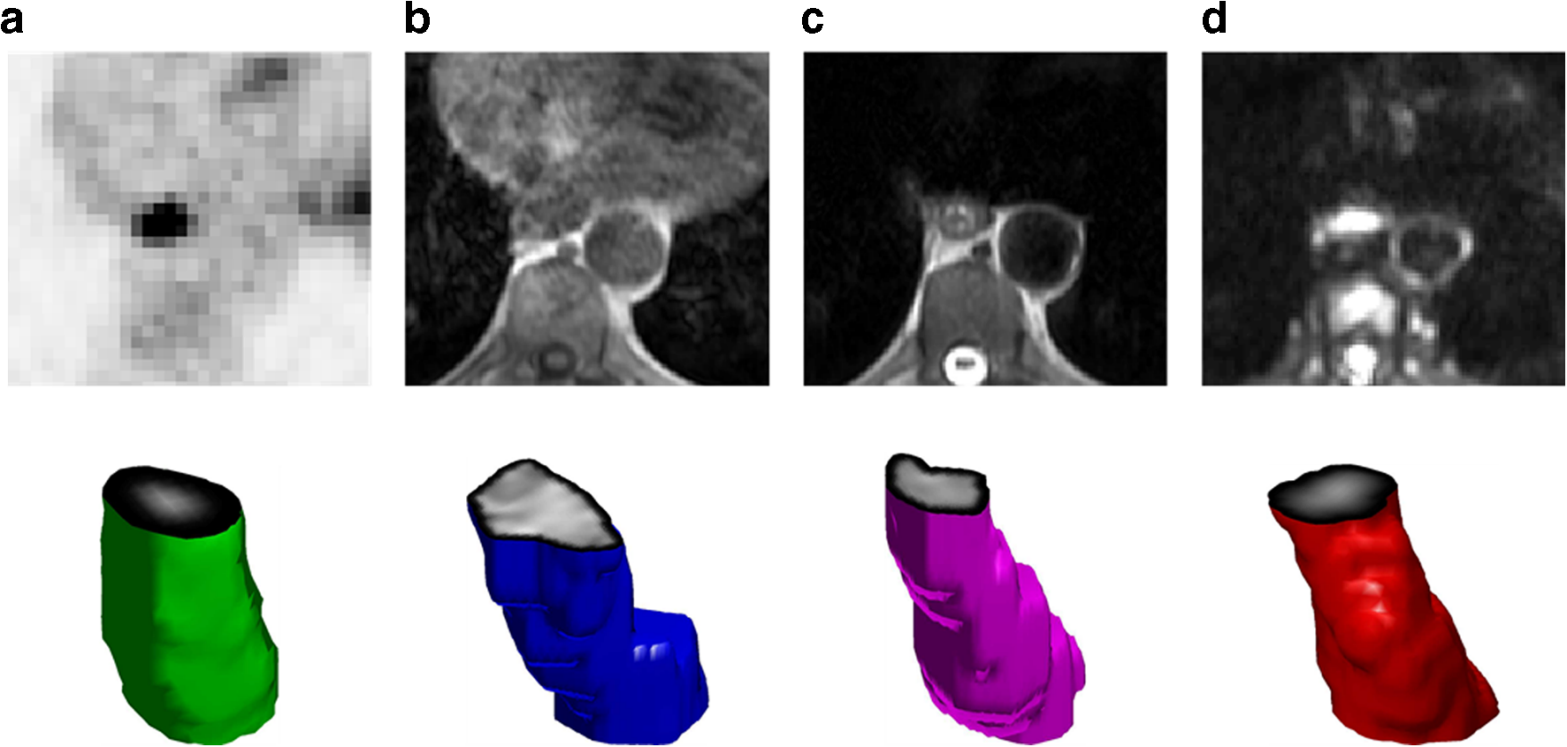
Axial ^18^F-FDG PET (**a**), T1-weighted (**b**), T2-weighted (**c**) and diffusion weighted (**d**) axial images of a GOJ cancer and respective tumour volumes

For each sequence investigated, segmentation of the whole tumour volume was performed manually by a dual-trained nuclear medicine physician/radiologist (with >5 years’ experience) through visual interpretation using ImageJ [https://imagej.nih.gov/ij/, [10]]. Feature generation and selection were performed using an in-house software based on MATLAB (MathWorks, Natick, MA, USA). First- (histogram) and second-order (grey-level co-occurrence matrix) statistical features (*n* = 24, Table 1) were computed for PET standardized uptake value (SUV) and MRI T1-W, T2-W, diffusion weighted and apparent diffusion coefficient (ADC) images from the whole tumour volume (Fig. 1). The maximum tumour diameter, defined as the maximum axis length of the tumour volume, was also measured.

**Table 1:** List of the first and second order features investigated

Order	Description	Features
First-order	Global analysis based on histogram intensity values	1. Mean 2. Standard deviation 3. Median 4. Minimum 5. Maximum 6. 10th percentile 7. 90th percentile 8. Entropy 9. Uniformity 10. Kurtosis 11. Skewness 12. Coefficient of variation
Second-order	Local analysis based on grey-level co-occurrence matrix (GLCM)	13. Entropy 14.Contrast 15. Homogeneity 16. Homogeneity normalized 17. Angular second moment 18. Joint maximum 19. Joint average 20. Joint variance 21. Inverse difference 22. Inverse difference normalized 23. Correlation 24. Autocorrelation

**Fig. 2:**
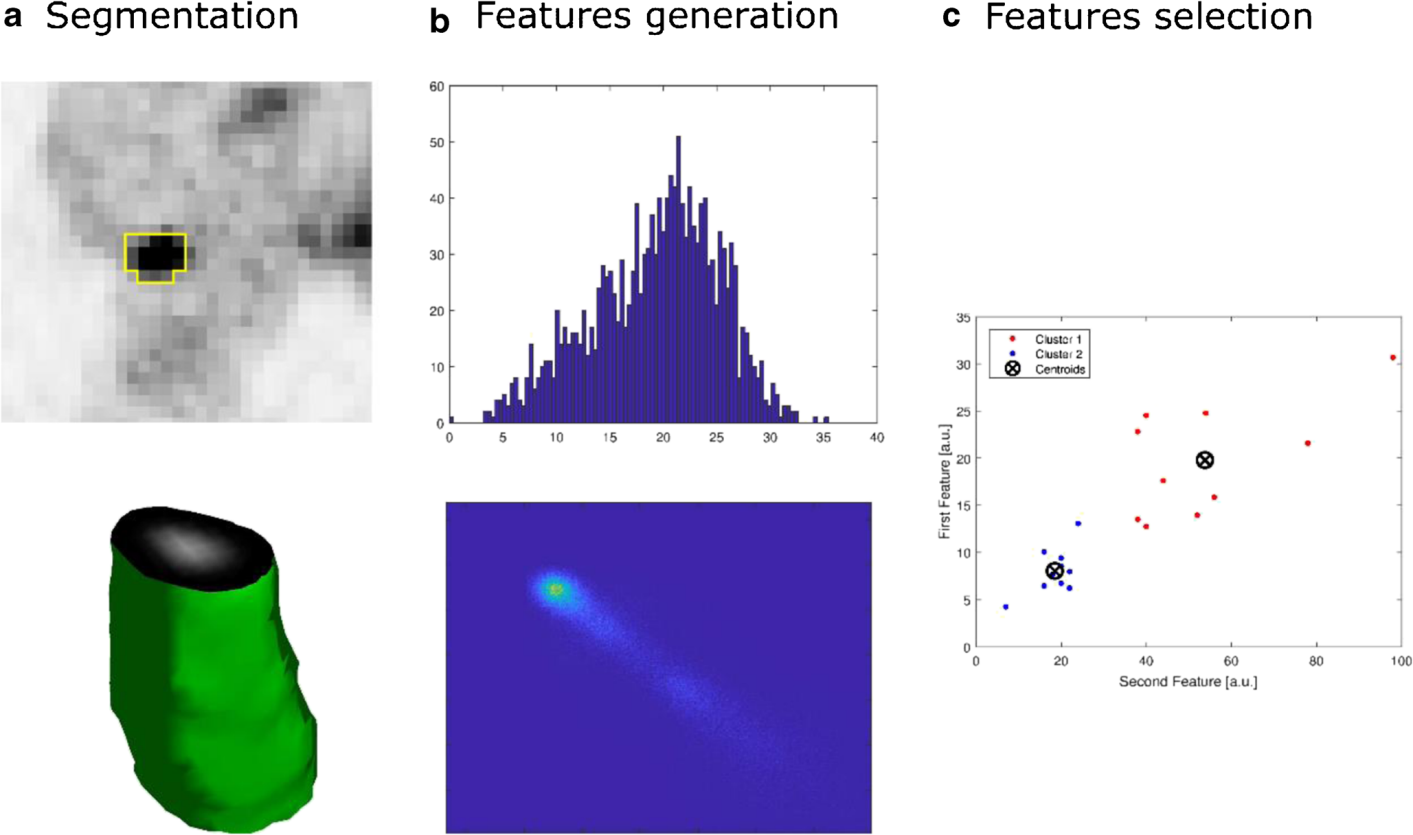
Schema demonstrating tumour segmentation (**a**) and subsequent feature generation (**b**) and selection (**c**) for ^18^F-FDG PET acquisition

Given the sample size, no more than two features were analysed jointly so as to minimize overfitting and spurious results. Feature selection started with a *k*-means (*k* = 2) clustering algorithm, an unsupervised classification method, which does not require a priori information (Fig. 2). This process iteratively identifies natural groupings, assigning feature-pairs values to the “nearest” class, maximizing the inter-class variance. Squared Euclidean distance was considered as similarity measure to determine the membership of the feature-pairs. Then, as a second step, the correlation of feature-pairs with the presence of metastases was analysed automatically a posteriori. Linear discriminant analysis (LDA) was used to determine the linear discrimination boundary.

### Statistical analysis

Multivariate analysis of variance (MANOVA) was performed to assess the statistical separability of the groups identified by feature-pairs (*p* < 0.001). Sensitivity (SN), specificity (SP), positive predictive value (PPV), negative predictive value (NPV) and accuracy (ACC) were calculated to quantify the discrimination ability of features in comparison with SUV_max_ ADC_mean_ and tumour size. In order to assess the potential impact that inter-observer variability might have on the feature reproducibility, the segmented tumour volumes were perturbed by performing automatic dilation and erosion, altering the lesion boundary by one pixel. Intraclass correlation coefficient (ICC) was computed by considering the feature values derived from dilated and eroded volumes. Statistical analysis was performed on MATLAB© (MathWorks, Natick, MA, USA).

## Results

Four patients were excluded for technical reasons (bulk motion, DIXON fat-water swap, poor quality diffusion imaging) precluding radiomic analysis, leaving 20 patients (18 male, 2 female, median age 67 years, range 52–86 years). Their tumour characteristics are summarised in Table 2. Ten patients had evidence of metastases including liver [3], lung [2], bone [1], and distant lymph nodes, e.g. retroperitoneal [6].

**Table 2:** Summary of tumour characteristics as defined by multidisciplinary meeting and incorporating all standard imaging tests

Tumour characteristics		Number of occurrences, *n* (N = 20)
Tumour type	Adenocarcinoma	17
	Squamous carcinoma	3
T stage	T1/2	0
	T3/4	20
N stage	Node negative	2
	Node positive	18
M stage	Non metastatic	10
	Metastatic	10

In terms of radiomic analysis, second-order GLCM entropy derived from SUV and ADC were the best feature-pair for discriminating patients with and without metastases (SN = 80%, SP = 80%, PPV = 80%, NPV = 80%, ACC = 80%). In particular, combined lower GLCM entropy derived from SUV and higher GLCM entropy from ADC, reflecting higher parameter spatial homogeneity and heterogeneity, respectively, was associated with metastatic disease. LDA proved the two groups identified by the clustering were linearly separated. The equation for the optimal separation of patients with and without metastatic disease is

$$ K+{L}_1{e}_{ADC}+{L}_2{e}_{SUV}=0 $$

where *K* = 40.90, *L*_*1*_ = −7.75, *L*_*2*_ = 6.25, *e*_*ADC*_ and *e*_*SUV*_ are the second-order entropy from ADC and SUV, respectively.

Patients with metastatic disease belonged to the half-plane given by the following inequality (Supplementary Figure 1):

$$ K+{L}_1{e}_{ADC}+{L}_2{e}_{SUV}<0 $$

MANOVA confirmed that the means of these features for the two groups of patients differed significantly, with a *p* value <0.001. In comparison, SUV_max_ (SN = 30%, SP = 80%, PPV = 60%, NPV = 53%, ACC = 55%), ADC_mean_ (SN = 20%, SP = 70%, PPV = 40%, NPV = 47%, ACC = 45%) and maximum tumour diameter alone (SN = 10%, SP = 90%, PPV = 50%, NPV = 50%, ACC = 50%) had poorer sensitivity and accuracy.

As far as the variability analysis was concerned, computed dilation and erosion altered the PET volumes by 34.8 ± 8.1 and 30.6 ± 6.2%, respectively. PET feature reproducibility errors for dilation and erosion were only 3.4 ± 3.0 and 3.1 ± 2.3%, respectively. Analogously, while ADC volume variations for dilation and erosion were 14.6 ± 2.8 and 13.9 ± 2.5%, respectively, feature reproducibility errors were 2.5 ± 1.1 and 3.1 ± 1.8%, respectively. The ICC for ADC and SUV GLCM entropy derived from dilated volumes were 0.96 and 0.98, respectively, while ICC derived from eroded volumes were 0.94 and 0.98, indicating the selected features were highly reproducible.

## Discussion

Better patient stratification remains a key challenge for the optimal management of GOJ patients. Our preliminary results indicate that pairing ^18^F-FDG PET and MRI radiomic features may highlight underlying GOJ biological heterogeneity associated with contemporaneous metastatic disease. In particular the *combination* of lower SUV second-order GLCM entropy and higher ADC GLCM entropy, which represents lower and higher local voxel heterogeneity (or irregularity), respectively, had a sensitivity and specificity of 80% for the presence of metastatic disease. Sensitivity was higher than SUV_max_, ADC_mean_ or maximum tumour diameter alone where sensitivity was 30, 20 and 10%, respectively.

Tissue accumulation of ^18^F-FDG is typically *increased* in most cancers including GOJ cancers, related to overexpression of cell-surface glucose transporters and increased hexokinase activity [11]. To date ^18^F-FDG PET/CT prognostic studies using SUV_max_ alone have reported mixed findings: one study found a lower SUV_max_ in patients with metastatic versus locally advanced primary tumours [12]; while another study found higher SUV_max_ in node positive versus node negative disease [13]. Other studies have found higher SUV_max_ (>3.5) in poorer outcome early cancers [14] and higher SUV_max_ in poor responders to CRT at 1 year [15]. However, a recent meta-analysis (*n* = 798) has suggested that SUV_max_ per se had no prognostic significance [16].

^18^F-FDG PET/CT radiomic studies have attempted to address this with mixed results. In terms of prognosis, one study (*n* = 406, adenocarcinoma and squamous carcinomas, varied therapies) found that lower first-order histogram energy, higher kurtosis and total lesion glycolysis (TLG) were associated with poorer overall survival [17]. However, another study by Nakajo et al. (*n* = 52) [18] found no association between radiomic features and progression-free and overall survival, although this study highlighted greater intensity and size zone variability in non-responders to chemoradiation.

Other radiomic studies have also found that various signatures are predictive of therapy response. Tixier et al. (*n* = 41, including adenocarcinoma and squamous carcinoma) showed that higher grey-level co-occurrence matrix (GLCM) homogeneity, lower grey-level size zone matrix (GLSZM) size zone variability and run length matrix (RLM) intensity variability differentiated responders from non-responders with sensitivities of 76–92% [19]. Beukinga et al. (*n* = 97, including adenocarcinoma and squamous carcinoma) found a higher PET-derived grey-level run length (GLRL) long run low grey-level emphasis in complete responders, and a clinical model including GLRL long run low grey-level emphasis had a higher area under the receiver operator curve (AUROC) compared to SUV_max_ alone [20]. Similarly, Van Rossum et al. (*n* = 217, adenocarcinoma) found reduction in RLM run percentage, GLCM entropy, and post-chemoradiation roundness improved prediction of response [21].

In contrast to higher FDG uptake, diffusion of water molecules is typically *reduced* in most cancers, again allowing cancers to be detected and effects of therapy to be monitored [22]. ADC represents the observed diffusion of water molecules, calculated from the slope of signal attenuation plotted against b-values. Of note, ADC is also influenced by factors including microscopic perfusion, bulk motion, acquisition sequence parameters and tissue orientation [23]. ADC_mean_ is the most commonly produced parameter and has good repeatability [24]. Few diffusion MRI studies have been performed for GOJ cancer to date with variable findings. A higher early change in ADC_mean_ [25] and higher change in ADC_75th percentile_ [26] has been noted in responders versus non-responders undergoing chemoradiation while another study found no association between ADC histogram parameters and therapy response [27].

Some studies have assessed if tumour size provides additional prognostic information. A study of squamous carcinoma (*n* = 387) found that small tumour size was an independent predictor of good outcome; the addition of tumour size to the AJCC TNM staging improved the predictive accuracy of the 5-year survival rate by 3.9% [28]. Studies of metabolic tumour volume have also found that low volume tumours have a better prognosis [29, 30]. In our study maximum tumour size had a low sensitivity for metastatic disease.

To date no studies have assessed the potential value of combining staging SUV and ADC parameters to provide insight into the tumour phenotype although the association between SUV_max_ and ADC_mean_ has been investigated in one study (*n* = 76, including adenocarcinoma and squamous carcinoma), showing no significant correlation [31].

The majority (85%) of our tumours were adenocarcinomas. These gland-forming tumours demonstrate a tubular, tubulopapillary or papillary growth pattern. Mucinous differentiation may be present in a small subset. Well-differentiated tumours show more than 95% gland formation while poorly differentiated tumours show <50% gland formation and are a more aggressive phenotype. Our metastatic signature of greater SUV local homogeneity combined with higher ADC heterogeneity is promising. We propose the greater homogeneity of SUV uptake on a local level relates to higher cellular versus stromal volume, i.e. more tightly packed predominantly FDG-avid tumour cells produce a more homogeneous tracer distribution. Greater local ADC heterogeneity likely reflects the varying glandular content, differentiation and mucinous content of tumours, i.e. greater heterogeneity represents a more aggressive histological subtype.

Nevertheless, there are a number of limitations to this study. Firstly, this was an exploratory prospective study and the number of patients was limited to 24, of which only 20 proceeded for further radiomic analysis. Secondly, while our findings of an association with contemporaneous metastases are promising, with a false positive rate of only 20%, this requires further study, including its potential as a future predictive or prognostic biomarker. Thirdly, assessment of progression free or overall survival was not undertaken due to our small sample size. Fourthly, the timing of PET/MRI post injection in our study was longer than 60 min. The impact of scan timing on GOJ PET radiomic features is unknown though a previous study including GLCM entropy is reassuring [32]. Finally, although this was a simultaneous acquisition using an integrated PET/MRI scanner, some spatial mismatch between PET and MRI data cannot be excluded given the location, and cardiac and respiratory motion.

In summary, a combined radiomic approach has the potential to improve risk stratification in GOJ cancer. Quantitative combined ^18^F-FDG PET and MRI features of the primary tumour from simultaneous PET/MRI scans are associated with a metastatic phenotype and may, in the future, help identify patients who will benefit from alternative therapeutic strategies or closer surveillance.

## Electronic supplementary material

ESM 1 (DOCX 86 kb)
