# Supplementary material for: Exploratory radiomic features from integrated 18F-fluorodeoxyglucose positron emission tomography/magnetic resonance imaging are associated with contemporaneous metastases in oesophageal/gastroesophageal cancer
Source: Eur J Nucl Med Mol Imaging. 2019 Mar 27;46(7):1478–84. doi: 10.1007/s00259-019-04306-7 (PMC6533412; doi:10.1007/s00259-019-04306-7)
Supplement: Supplementary file 1 — (DOCX 86 kb) [file 259_2019_4306_MOESM1_ESM.docx]

**Supplemental Data**

Supplemental Figure 1. Distribution of GLCM entropy from ADC and SUV for patients with metastatic disease (M1, highlighted in red) and no metastatic disease (M0, highlighted in blue).


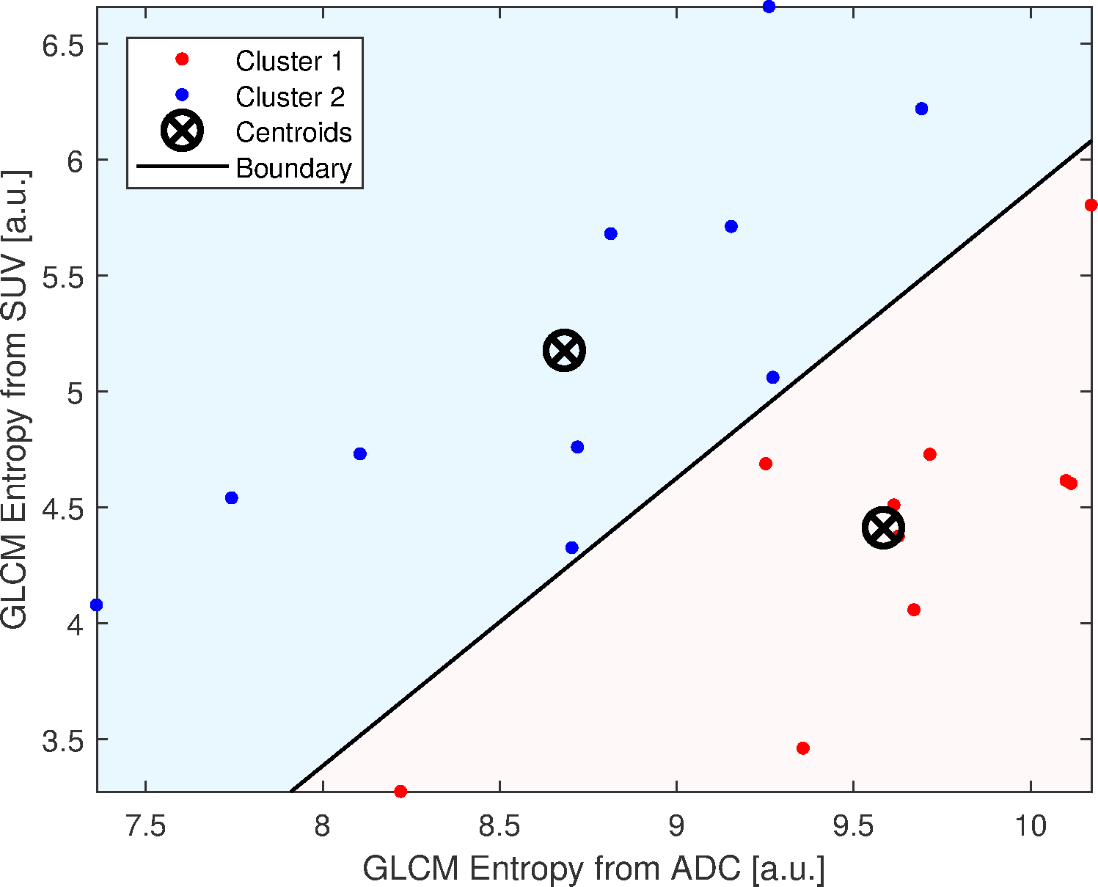


GLCM entropy from ADC and SUV can discriminate patients only if used jointly. Centroids represent features mean values of the two groups identified by the clustering (marked with black crossed circle).

The linear discrimination boundary (highlighted in black) represents the set of points where the two groups are equally probable. It is given by the following equation

$$K+L_{1}e_{ADC}+L_{2}e_{SUV}=0$$

where *K* = 40.90, *L_1_* = -7.75, *L_2_* = 6.25, *e_ADC_* and *e_SUV_* are the GLCM entropy from ADC and SUV, respectively.

The presence of metastasis is associated with a higher entropy of the ADC map, i.e. higher heterogeneity, along with a lower entropy of the SUV map, i.e. higher homogeneity. This is formally expressed by the following inequality, representing the half plane highlighted in pink:

$$K+L_{1}e_{ADC}+L_{2}e_{SUV}<0$$

The absence of metastasis is associated with simultaneous presence of “homogeneity” on the two maps, i.e. low values for both features, or to the presence of heterogeneity on the SUV map. This is formally expressed by the following inequality, representing the half plane highlighted in light blue:

$$K+L_{1}e_{ADC}+L_{2}e_{SUV}>0$$
